# pH and Magnetism Dual-Responsive Pickering Emulsion Stabilized by Dynamic Covalent Fe_3_O_4_ Nanoparticles

**DOI:** 10.3390/nano12152587

**Published:** 2022-07-28

**Authors:** Gaihuan Ren, Zhanzhao Li, Dongxu Lu, Bo Li, Lulu Ren, Wenwen Di, Hongqin Yu, Jianxin He, Dejun Sun

**Affiliations:** 1 Textile and Garment Industry of Research Institute, Zhongyuan University of Technology, Zhengzhou 450007, China; 2021117608@zut.edu.cn (Z.L.); 2020110366@zut.edu.cn (B.L.); 3812@zut.edu.cn (H.Y.); 5269@zut.edu.cn (J.H.); 2 School of Mechanical and Electrical Engineering, Zhengzhou University of Industrial Technology, Zhengzhou 450007, China; tian543669@163.com; 3 Key Laboratory of Colloid and Interface Chemistry, Ministry of Education, Shandong University, Jinan 250100, China; 201912144@mail.sdu.edu.cn (L.R.); 201832281@mail.sdu.edu.cn (W.D.); djsun@zut.edu.cn (D.S.)

**Keywords:** Pickering emulsion, dynamic covalent bond, pH-responsive, magnetism-responsive, extraction efficiency

## Abstract

Herein, we describe pH and magnetism dual-responsive liquid paraffin-in-water Pickering emulsion stabilized by dynamic covalent Fe_3_O_4_ (DC-Fe_3_O_4_) nanoparticles. On one hand, the Pickerinfigureg emulsions are sensitive to pH variations, and efficient demulsification can be achieved by regulating the pH between 10 and 2 within 30 min. The dynamic imine bond in DC-Fe_3_O_4_ can be reversibly formed and decomposed, resulting in a pH-controlled amphiphilicity. The Pickering emulsion can be reversibly switched between stable and unstable states by pH at least three times. On the other hand, the magnetic Fe_3_O_4_ core of DC-Fe_3_O_4_ allowed rapid separation of the oil droplets from Pickering emulsions under an external magnetic field within 40 s, which was a good extraction system for purifying the aqueous solution contaminated by rhodamine B. The dual responsiveness enables Pickering emulsions to have better control of their stability and to be applied more broadly.

## 1. Introduction

Pickering emulsions, which were stabilized by solid particles, have gained much attention thanks to the low toxicity and long-term stability [1,2]. The outstanding stability of Pickering emulsions is required in the field of cosmetic formulations, food storage, and so on [3]. However, in some fields like heterogeneous catalysis [4,5], emulsion polymerization [6], and oil transportation and recovery [7,8,9], temporary stabilization is required. Chemical or physical methods are used in the industry to achieve demulsification, which are often energy-intensive or require extra complex additives, leading to increased economic and environmental costs [10,11]. Thus, stimuli-responsive Pickering emulsions are desired in the above applications because the stability of the emulsions can be easily controlled by external stimuli [12].

Recently, stimuli-responsive Pickering emulsions have received much attention [13,14,15,16,17]. So far, the stimuli of the Pickering emulsions include pH [18,19,20], magnetism [21,22,23], CO_2_ [24], temperature [25], light irradiation [26], redox state [27], specific ion concentration [28], and so on. Under outside stimuli, stable Pickering emulsions can be phase separated because of the inactivation of the stabilizer. There is merit in combining two stimuli to widen the controllable range or improve the degree of precision [16]. Pickering emulsions responding to dual triggers such as temperature–pH [29], CO_2_–temperature [16], CO_2_–magnetism [14,30], and redox–magnetism [9] have been investigated. For CO_2_-stimulated systems, a long time and high ventilation are needed [31]. Light-stimulated systems are difficult to achieve because of the cloudy appearance of the Pickering emulsions [32]. Temperature-stimulated systems are energy-demanding [31]. Among these stimuli, pH is readily implementable and magnetism is non-invasive and reversible [2,7,10,32,33]. Therefore, pH and magnetism dual-responsive Pickering emulsions have promising applications.

Magnetism-responsive Pickering emulsions have received considerable attention because of their application as extractors for dye molecules, such as Rhodamine B, methylene blue, and Nile blue [9]. Yang et al. [34] prepared a kind of magnetism-responsive Pickering emulsion using amphiphilic Fe_3_O_4_ particles as a stabilizer, which could be used as an extractor to remove methyl orange from the aqueous solution. Therefore, magnetism-responsive Pickering emulsion can be an attractive tool to remove dye molecules in water.

In the present study, dynamic covalent Fe_3_O_4_ (DC-Fe_3_O_4_) was prepared through pH sensitive dynamic imine bond (DIB) formation between amino-Fe_3_O_4_ (Fe_3_O_4_-NH_2_) and benzaldehyde. The amphiphilicity of DC-Fe_3_O_4_ nanoparticles could be modulated by pH because of the pH-responsiveness of DIB. The prepared amphiphilic DC-Fe_3_O_4_ could achieve effective emulsification of the oil phase at pH 10, while the following demulsification process could be completed by decreasing the pH from 10 to 2. Moreover, the droplets move in the direction of the magnet. The magnetism-responsive Pickering emulsion could be used as an extractor to adsorb Rhodamine B (RhB) from aqueous solution at least three times. This novel pH and magnetism dual-responsive Pickering emulsion has potential applications in oil recovery, emulsion polymerization, and dye extraction.

## 2. Experimental

### 2.1. Materials

Iron sulfate heptahydrate (FeSO_4_·7H_2_O, AR), ferric chloride (FeCl_3_, AR), sodium hydroxide (NaOH, AR), (3-aminopropyl) triethoxysilane (APTES, 98%), Nile red (≥95.0%), and Rhodamine B (RhB, AR) were obtained from Aladdin Reagents of China. Benzaldehyde (AR), liquid paraffin (AR), and hydrogen chloride (36.5 wt%) were supplied by Sinopharm Chemical Reagent Co. Ltd., Shanghai, China. All of the chemicals were used as received without any further purification.

### 2.2. Synthesis of DC-Fe_3_O_4_ Nanoparticles

Fe_3_O_4_ was synthesized using the co-precipitation method [35]. FeSO_4_ solution with a concentration of 0.5 M and FeCl_3_ solution with a concentration of 1 M were prepared using 0.2 M HCl (aq), respectively, as solvent. During preparation, 400 mL of 1.5 M NaOH (aq) was poured into the three flasks and heated to 82 °C, and then the mixture of FeSO_4_ solution (40 mL, 0.5 M) and FeCl_3_ solution (40 mL, 1 M) was added to the three flasks dropwise within 30 min under the atmosphere of N_2_ at 82 °C. As soon as the mixture turned to black, it was allowed to cool down to 25 °C under mechanical stirring. Then, the resultant black nanoparticles were washed with ethanol four times with the help of a magnet. The obtained product Fe_3_O_4_ nanoparticles were dried under a vacuum for 16 h.

**Figure 1:**
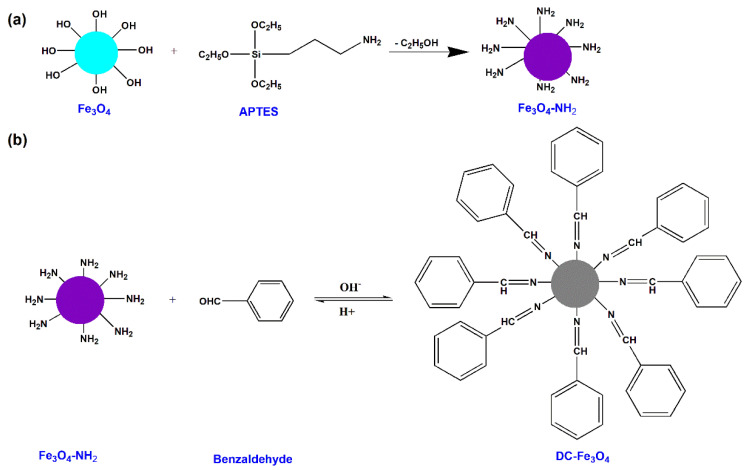
Schematic illustration of the synthesis of Fe_3_O_4_-NH_2_ (**a**) and DC-Fe_3_O_4_ (**b**).

The amino modified Fe_3_O_4_ (Fe_3_O_4_-NH_2_) nanoparticles were obtained by surface silanization of Fe_3_O_4_ nanoparticles (Figure 1a). In detail, 0.5 mL APTES and 0.95 g Fe_3_O_4_ were added into 25 mL ethanol at 25 °C. After stirring for 24 h, the obtained Fe_3_O_4_-NH_2_ was washed with ethanol four times with the help of a magnet and then dried under vacuum for 16 h.

To prepare dynamic covalent Fe_3_O_4_ (DC-Fe_3_O_4_), Fe_3_O_4_-NH_2_ (1.0 g) and benzaldehyde were mixed in methanol (20 mL) under mechanical stirring for 1 h; the molar ratio of -NH_2_ and -CHO was 1. A schematic illustration of the synthesis of DC-Fe_3_O_4_ is presented in Figure 1b. The product was collected and then washed with ethanol four times with the help of a magnet. The product DC-Fe_3_O_4_ nanoparticles were dried under vacuum for 16 h.

### 2.3. Preparation of Pickering Emulsions

For Pickering emulsions’ preparation, water, DC-Fe_3_O_4_ nanoparticles, and liquid paraffin were placed into a glass bottle. The concentration of DC-Fe_3_O_4_ varied from 0.1 to 2.0 wt%. The water and liquid paraffin were in an equal volume ratio. Then, the mixture was homogenized using a JY88-IIN sonicator with a 6 mm probe at 50 W twelve times (5 s, 5 s off) to obtain Pickering emulsions. The Pickering emulsions were kept at room temperature for 1 month to observe the storage stability.

### 2.4. Characterization of DC-Fe_3_O_4_ Nanoparticles

DC-Fe_3_O_4_ nanoparticles were dispersed in water at a concentration of 0.05 wt%. Approximately 20 μL of the above DC-Fe_3_O_4_ dispersion was loaded onto a copper grid. The DC-Fe_3_O_4_ sample was allowed to dry and imaged using a transmission electron microscope (Hitachi HT7700).

The composition of the Fe_3_O_4_, Fe_3_O4-NH_2_, and DC-Fe_3_O_4_ was investigated by FTIR spectrometer (Bruker Optics, Germany). The morphology of DC-Fe_3_O_4_ was characterized using TEM (Hitachi HT7700). For solid/air/water three-phase contact angle measurement, samples of the Fe_3_O_4_, Fe_3_O_4_-NH_2_, and DC-Fe_3_O_4_ were compressed into films.

For three-phase (solid/air/water) contact angle measurements, samples of the Fe_3_O_4_, Fe_3_O_4_-NH_2_, and DC-Fe_3_O_4_ nanoparticles were compressed into films using a table press (Shimadzu Press). The contact angle was measured using the contact angle goniometer (DataPhysics Instruments GmbH, Filderstadt, Germany). The contact angle was recorded by placing a droplet of water with a volume of 3.0 μL onto the film in the air. When the water was in contact with the film, the image of the morphology of the water droplet on the film surface was recorded and later analyzed by the software Photoshop to obtain the contact angle.

### 2.5. Characterization of Pickering Emulsions

To conform the emulsion type, liquid paraffin was stained with Nile red. The Pickering emulsion droplets were detected with a confocal fluorescence microscope (Carl Zeiss, Oberkochen, Germany). The micrographs of the Pickering emulsions were observed with an A1Pol optical microscope (ZEISS, Oberkochen, Germany).

### 2.6. pH Modulation of Pickering Emulsion

The pH modulation (between pH 10 and 2) of the Pickering emulsion was achieved by alternately adding 1 M HCl or 1 M NaOH, followed by stirring for 30 s and standing for 30 min.

### 2.7. Magnetic Modulation of Pickering Emulsion

The magnetism-responsive character of the Pickering emulsion was carried out by applying an NdFeB permanent magnet (Jiangsu Lingxi Magnetic Industry Co., Suzhou, China) with a size of 60 × 40 × 5 mm.

### 2.8. Extraction of RhB from the Aqueous Solution

The RhB-polluted aqueous solution (4 mg/L) was prepared by dissolving RhB in water. Pickering emulsion (1 mL) was added to RhB-polluted aqueous solution (5 mL) to extract RhB. After standing for 20 min, the purified water was separated over a magnet and poured out. Prior to and after extraction, the concentration of RhB was determined using a UV/Vis spectrophotometer at 553 nm. According to the following equation, the extraction efficiency (E) can be calculated:

(1) $$E=\frac{C_{0}{-C}_{e}}{C_{0}}$$

where C_0_ (mg/L) and C_e_ (mg/L) are the concentrations of RhB aqueous solution before and after extraction, respectively. In addition, Pickering emulsion could be obtained again by washing with water, and the extraction process could be repeated at least three times.

## 3. Results and Discussion

### 3.1. Characterization of Dynamic Covalent Fe_3_O_4_

**Figure 2:**
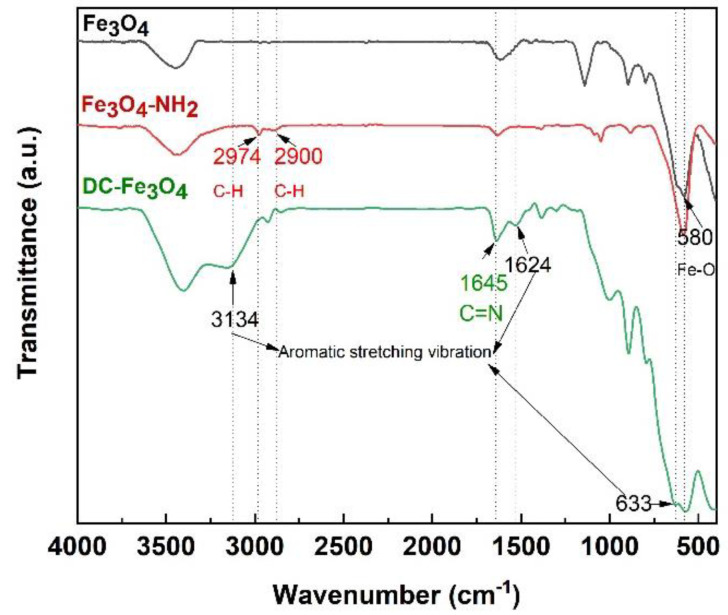
FTIR spectra of Fe_3_O_4_, Fe_3_O_4_-NH_2_, and DC-Fe_3_O_4_ nanoparticles.

The preparation of DC-Fe_3_O_4_ nanoparticles through DIB formation between amino-Fe_3_O_4_ (Fe_3_O_4_-NH_2_) and benzaldehyde is presented in Figure 1. FTIR was performed to prove the successful fabrication of DC-Fe_3_O_4_ nanoparticles. As shown in Figure 2, the bond at 580 cm^−1^ in the Fe_3_O_4_ FTIR spectra was the characteristic peak of the Fe-O functional groups. Just like the peak presented in Fe_3_O_4_, the Fe-O absorption peak (580 cm^−1^) also appears in the spectra of Fe_3_O_4_-NH_2_ and DC-Fe_3_O_4_, suggesting the presence of Fe_3_O_4_ in Fe_3_O_4_-NH_2_ and DC-Fe_3_O_4_. The FTIR spectrum of Fe_3_O_4_-NH_2_ shows sp^3^ C-H asymmetric and symmetric stretching vibration at 2974 and 2900 cm^−1^, respectively, demonstrating the presence of aminopropyl groups in Fe_3_O_4_-NH_2_. For DC-Fe_3_O_4_, the presence of aromatic stretching vibrations at 3134, 1624, and 633 cm^−1^, as well as the imine bond (C=N) stretching vibration at 1645 cm^−1^, confirmed the formation of DC-Fe_3_O_4_ between Fe_3_O_4_-NH_2_ and benzaldehyde via DIB. The morphology DC-Fe_3_O_4_ nanoparticles were characterized by TEM (Figure S1). The TEM image shows that the DC-Fe_3_O_4_ nanoparticles have a nearly spherical shape with a mean diameter of about 17 nm. The DC-Fe_3_O_4_ nanoparticles exhibit an instant magnetic response to the external magnetic field and can be separated completely from their liquid dispersions within 30 min. When the magnetic field is removed, the DC-Fe_3_O_4_ nanoparticles can be dispersed again by shaking.

**Figure 3:**
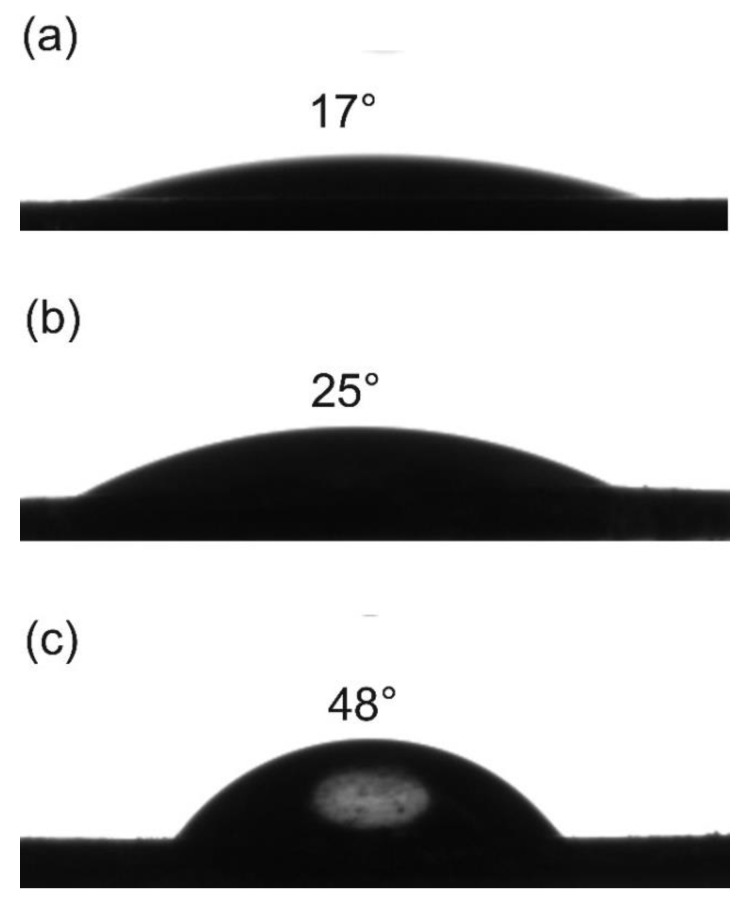
Contact angles of water on Fe_3_O_4_ film (**a**), Fe_3_O_4_-NH_2_ film (**b**), and DC-Fe_3_O_4_ film (**c**).

The stabilization of two immiscible phases was related to the wettability of particles at their interface, which was measured by contact angle [36,37,38]. Therefore, contact angle measurement was carried out for Fe_3_O_4_, Fe_3_O_4_-NH_2_, and DC-Fe_3_O_4_, respectively. Fe_3_O_4_ was found to have a contact angle of roughly 17° (Figure 3a), which was too hydrophilic for stabilizing Pickering emulsions; a similar result was reported by Sun et al. [9]. When Fe_3_O_4_ nanoparticles were modified by APTES, Fe_3_O_4_-NH_2_ nanoparticles also exhibit a hydrophilic character with a contact angle of about 25° (Figure 3b), which was also too hydrophilic for stabilizing Pickering emulsions. By modifying Fe_3_O_4_-NH_2_ nanoparticles with relatively hydrophobic benzaldehyde through DIB formation, the contact angle of modified Fe_3_O_4_ nanoparticles (DC-Fe_3_O_4_) increased to about 48° (Figure 3c). The improved amphiphilicity of DC-Fe_3_O_4_ is desirable to the formation of stable Pickering emulsions. It is well-known that the particle contact angle determines the type of Pickering emulsion [39,40]. If the contact angle of particles is less than 90°, they are located preferentially in the water phase, and the resulting curvature favors O/W Pickering emulsions. In contrast, if the contact angle exceeds 90°, the particles will reside primarily in the oil phase, which will lead to W/O Pickering emulsions [41]. Hence, amphiphilic DC-Fe_3_O_4_ nanoparticles with a contact angle of about 48° are expected to prepare O/W Pickering emulsions.

**Figure 4:**
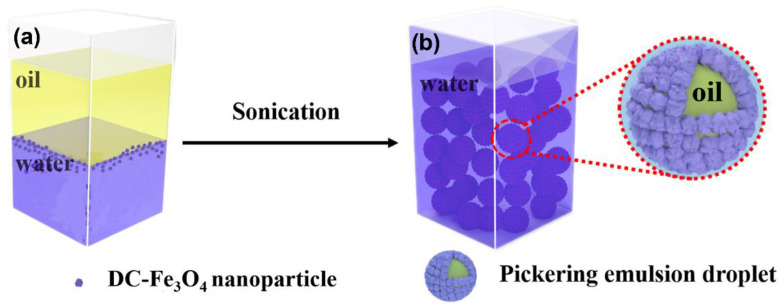
Preparation process of Pickering emulsion stabilized by DC-Fe_3_O_4_ with a volume ratio of liquid paraffin and water of 1:1. (**a**) The mixture of liquid paraffin, water and DC-Fe_3_O_4_ nanoparticles before sonication, (**b**) the obtained Pickering emulsions after sonicating the mixture of liquid paraffin, water and DC-Fe_3_O_4_ nanoparticles in (**a**).

The amphiphilicity of DC-Fe_3_O_4_ can be further proved by the dispersion behavior of DC-Fe_3_O_4_ on the liquid paraffin–water two phases. The DC-Fe_3_O_4_ nanoparticles straddle on the liquid paraffin–water interface instead of the aqueous phase even after shaking (Figure S2 and Figure 4a), indicating the amphipathic nature of the DC-Fe_3_O_4_ nanoparticles, consistent with the contact angle measurement.

### 3.2. Preparation of Pickering Emulsions Stabilized by DC-Fe_3_O_4_ Nanoparticles

The liquid paraffin-in-water Pickering emulsion could not be stabilized by Fe_3_O_4_ or Fe_3_O_4_-NH_2_ alone for 30 min at the particle concentration of 1.0 wt% (a rather high concentration in Pickering emulsion preparation, Figure S3). The reason that Fe_3_O_4_ or Fe_3_O_4_-NH_2_ nanoparticles could not be used as the Pickering emulsifier might be attributed to the high hydrophilicity of Fe_3_O_4_ and Fe_3_O_4_-NH_2_, as proven by the result of the contact angle (Figure 3).

**Figure 5:**
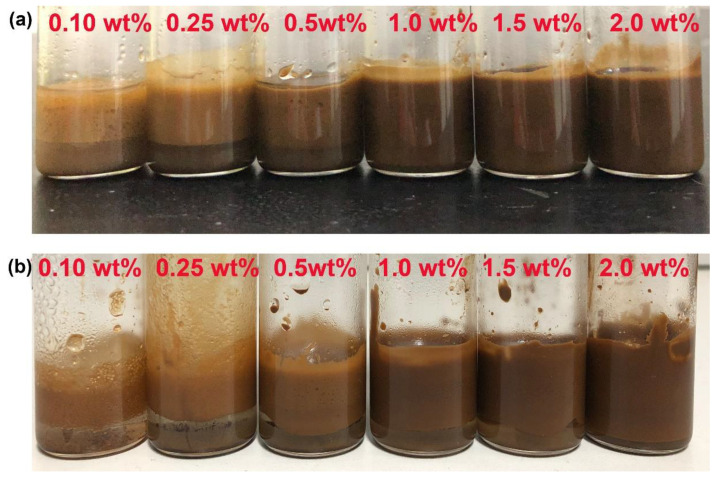
Photographs of the oil-in-water Pickering emulsions stabilized by DC-Fe_3_O_4_ nanoparticles at different concentrations. The photographs were taken at 24 h (**a**) and 1 month (**b**) after the sample preparation. The liquid paraffin and water were in an equal volume ratio. Concentrations of DC-Fe_3_O_4_ (wt%) in the Pickering emulsions are shown on the vessels.

**Figure 6:**
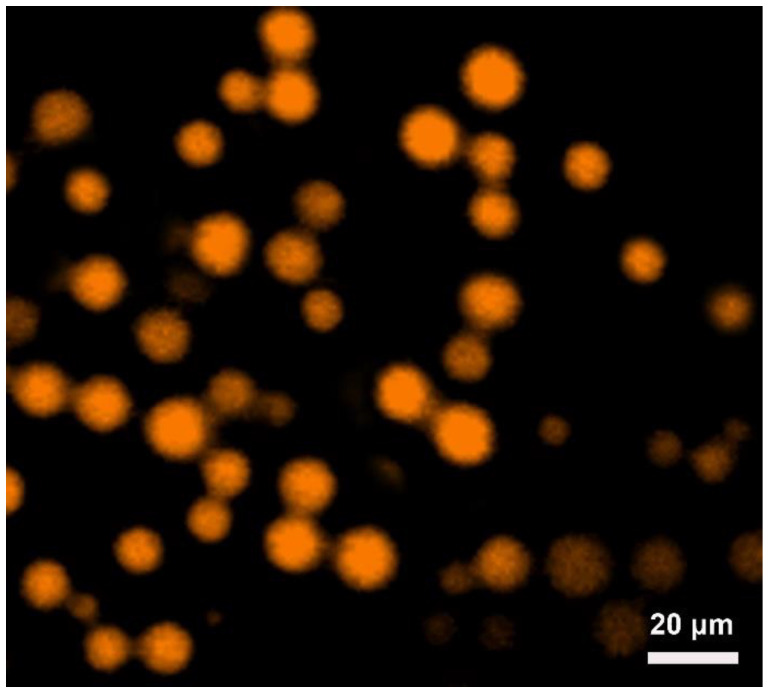
CLSM image of 1.0 wt% DC-Fe_3_O_4_ nanoparticles stabilized liquid paraffin in water Pickering emulsion. The liquid paraffin (stained by Nile Red) and water were in an equal volume ratio.

Based on the discussion of the contact angle, we presume that the amphiphilic DC-Fe_3_O_4_ nanoparticles with a contact angle of about 48° are anticipated to prepare O/W Pickering emulsions. The ability of DC-Fe_3_O_4_ nanoparticles to stabilize the Pickering emulsions was investigated; the preparation process of Pickering emulsions is presented in Figure 4. In the control experiment, we have proved that no emulsion was prepared without DC-Fe_3_O_4_ nanoparticles (Figure S4). With 0.1 wt% DC-Fe_3_O_4_ nanoparticles, no homogeneous Pickering emulsion could be prepared because of insufficient DC-Fe_3_O_4_ nanoparticles on the oil–water interface (Figure 5). Satisfactorily, when the DC-Fe_3_O_4_ nanoparticle concentrations were equal to or exceeding 0.25 wt%, stable Pickering emulsions were prepared (Figure 5), which might due to the effective adsorption of amphiphilic DC-Fe_3_O_4_ nanoparticles at the liquid paraffin–water interface (Figure 4b). The CLSM measurement shows that the labeled liquid paraffin is surrounded by the unlabeled water (Figure 6), indicating that O/W Pickering emulsion was formed.

**Figure 7:**
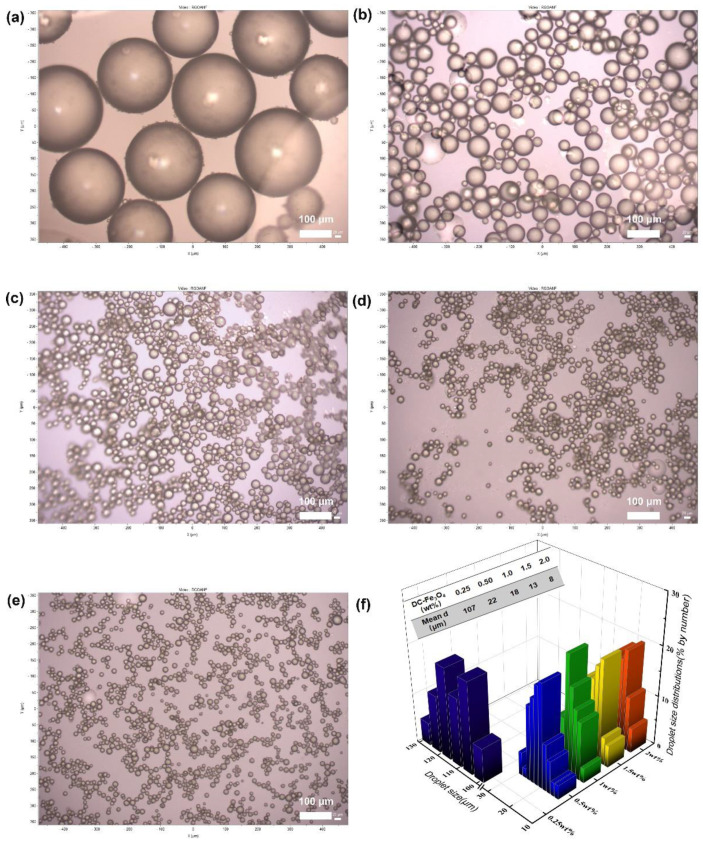
Optical microscopes of the oil-in-water Pickering emulsions stabilized by DC-Fe_3_O_4_ nanoparticles at different concentrations with an equal volume of liquid paraffin and water. From (**a**–**e**), the DC-Fe_3_O_4_ concentration is 0.25, 0.5, 1.0, 1.5, and 2.0 wt%, respectively. The images were taken at 24 h preparation. (**f**) Droplet size distribution of Pickering emulsion stabilized DC-Fe_3_O_4_ nanoparticles at different concentrations. The liquid paraffin and water were in an equal volume ratio.

The morphology of Pickering emulsions was observed by optical microscopy (Figure 7a–e). As shown in Figure 7a–e, small and spherical droplets were observed. According to the statistic, droplets became smaller with increasing DC-Fe_3_O_4_ nanoparticle concentrations: the mean droplet sizes of Pickering emulsions with a DC-Fe_3_O_4_ nanoparticle concentration of 0.25, 0.5, 1.0, 1.5, and 2.0 wt% were 107, 22, 18, 13, and 8 μm, respectively (Figure 7f). This was because more particles were able to stabilize larger interfaces at a constant oil–water ratio, which is a common feature for Pickering emulsions [10,17]. In this study, it was found that the size of Pickering emulsion droplets can be easily adjusted by choosing a suitable DC-Fe_3_O_4_ nanoparticle concentration.

**Figure 8:**
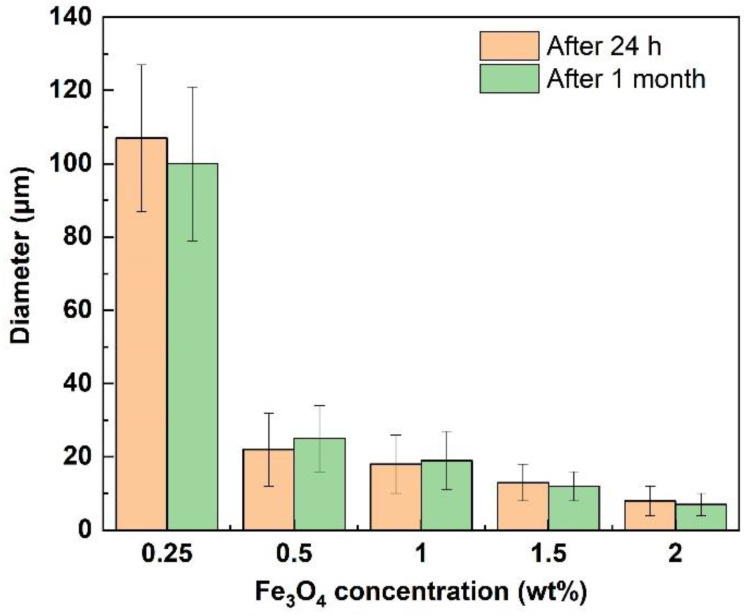
The mean droplet size of Pickering emulsions after 24 h and after 1 month of storage at different stabilized DC-Fe_3_O_4_ concentrations. The liquid paraffin and water were in an equal volume ratio.

The Pickering emulsions showed almost no change in appearance after one month of storage, and no clear oil phase could be observed (Figure 5a,b), indicating the high stability of Pickering emulsions. Meanwhile, even after 1 month of storage, the droplet size of the Pickering emulsions remained nearly unchanged (Figure 8), further confirming their long-term stability. It was found that Pickering emulsions were stable for a long time because of the irreversible adsorption of DC-Fe_3_O_4_ nanoparticles at the O/W interface.

### 3.3. pH-Responsive Behavior of the Pickering Emulsions

On the basis of the above discussions, we can conclude that stable Pickering emulsions were obtained using the amphiphilic DC-Fe_3_O_4_ nanoparticles as a stabilizer. Considering the dynamic character of DIB to pH [42,43,44,45,46], the amphiphilicity of DC-Fe_3_O_4_ nanoparticles may be changed by transforming pH. To verify this, contact angle measurement for the DC-Fe_3_O_4_ film with acidic water (pH 2) was conducted. The contact angle of the DC-Fe_3_O_4_ nanoparticle film decreased from about 48° to about 25° after adding water droplets with a pH of 2 (Figure S5), indicating the dissociation of amphiphilicity DC-Fe_3_O_4_ into hydrophilic Fe_3_O_4_-NH_2_ and benzaldehyde in the acidic environment. That is to say, the amphiphilicity of DC-Fe_3_O_4_ nanoparticles could be changed by changing the pH.

**Figure 9:**
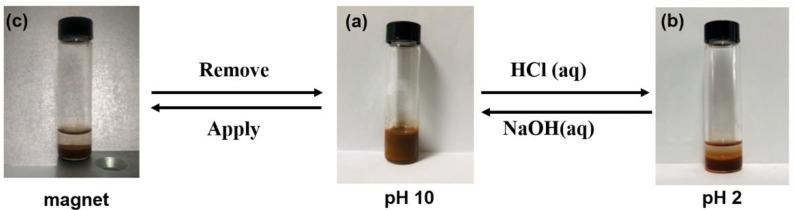
The pH and magnetism dual-responsive oil-in-water Pickering emulsion prepared by 1.0 wt% DC-Fe_3_O_4_ nanoparticles. The Pickering emulsion sample at pH 10 (**a**), after reducing the pH to 2 (**b**), under an external magnetic field (**c**).

**Scheme 1:**
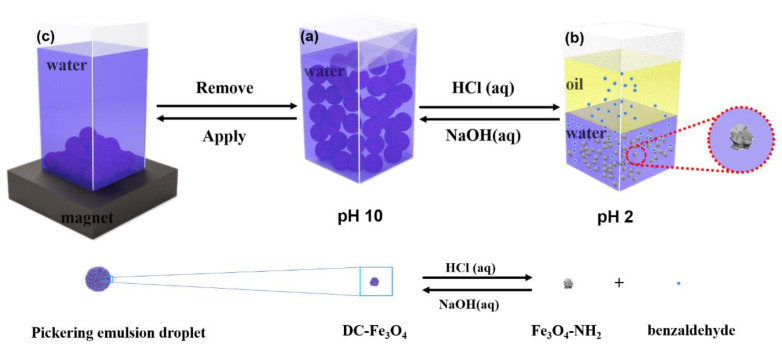
Illustration of pH and magnetism dual-responsive Pickering emulsion stabilized by DC-Fe_3_O_4_ nanoparticles. (**a**) Stable Pickering emulsion, (**b**) phase separated system after decreasing pH from 10 to 2 by adding 1 M HCl (1 M), and (**c**) Pickering emulsion droplets were collected by placing the magnet on the bottom of the bottle.

In light of the change in amphiphilicity of DC-Fe_3_O_4_ nanoparticles when the pH is changed, we speculated that the pH could be used to adjust the stability of the obtained Pickering emulsions. To validate this hypothesis, liquid paraffin-in-water Pickering emulsion stabilized by 1.0 wt% DC-Fe_3_O_4_ nanoparticles was used as the representative sample. At pH 10, DC-Fe_3_O_4_ nanoparticles can be used as a stabilizer to prepare O/W Pickering emulsions because of the effective adsorption of amphiphilic DC-Fe_3_O_4_ at the liquid paraffin–water interface (Figure 9a and Scheme 1a). By decreasing the pH from 10 to 2, complete phase separation was achieved within 30 min (Figure 9b and Scheme 1b). At pH 2, the amphiphilic DC-Fe_3_O_4_ nanoparticles decomposed into hydrophilic Fe_3_O_4_-NH_2_ and inactive benzaldehyde, both of which were desorbed from the oil–water interface, causing demulsification (Figure 9b and Scheme 1b). The hydrophilic Fe_3_O_4_-NH_2_ nanoparticles participated in the aqueous phase (Scheme 1b). Additionally, benzaldehyde is surface-inactive and cannot stabilize emulsions effectively, as reported by our previous study [17]. We estimate that about 53% of the benzaldehyde migrates into the liquid paraffin phase based on the UV/Vis results (Scheme 1b). Moreover, after increasing the pH from 2 to 10, stable Pickering emulsion was reformed after re-sonication because of the re-formation of amphiphilic DC-Fe_3_O_4_ nanoparticles through DBI formation between hydrophilic Fe_3_O_4_-NH_2_ and surface inactive benzaldehyde (Figure 9a and Scheme 1a). Furthermore, pH-induced reversible emulsification and demulsification can be repeated up to three times without loss of efficacy (Figure 9a,b and Scheme 1a,b). Additionally, after three emulsification and demulsification cycles, the size of a newly formed Pickering emulsion shows a negligible increase compared with the original Pickering emulsion (13.1 μm vs. 13.5 μm, Figure S6). Emulsification and demulsification of the Pickering emulsion are determined by the pH responsiveness of the imine bond.

### 3.4. Magnetism-Responsive Behavior of the Pickering Emulsions

According to the reports, Pickering emulsion droplets stabilized by Fe_3_O_4_ nanoparticles can move in the direction of a magnet, or even coalesce when subjected to magnetic fields [9,23]. Upon exposure to a magnet, the DC-Fe_3_O_4_-coated Pickering emulsion droplets showed an instantaneous response with unidirectional movement toward the magnet, as demonstrated in Figure 9c and Scheme 1c, which was attributed to the superparamagnetic property of DC-Fe_3_O_4_ (magnetic saturation value, 43 emu/g). The time needed to remove all of the droplets was 40 s.

Magnetism-responsive Pickering emulsions have been widely used in extracting organic pollutants from aqueous solutions [9,34]. Here, we discuss the possibility of using the magnetism-responsive Pickering emulsion to extract a pollutant. The pink color could be seen in photos of the RhB solution (Figure S7a). Nonetheless, the pink color in RhB aqueous solution nearly disappeared after Pickering emulsion was added for 20 min, which suggested that RhB molecules were efficiently removed from the aqueous solution (Figure S7d). Based on the standard curve for RhB solution (Figure S8), the extraction efficiency for RhB adsorption was 97.5% (Figure S9). A similar adsorption experiment was conducted using only DC-Fe_3_O_4_ nanoparticle aqueous dispersion or liquid paraffin as an adsorbent in order to explore the adsorption mechanism. After adding DC-Fe_3_O_4_ nanoparticle aqueous dispersion or liquid paraffin into RhB aqueous solution for 1 h, the pink color did not disappear (Figure S7b,c). Compared with DC-Fe_3_O_4_ nanoparticle aqueous solution or liquid paraffin, Pickering emulsions have a higher specific surface area. Therefore, the high specific surface area of Pickering emulsions played an important role in the adsorption of RhB from the water phase into the oil–water interface. Besides, by replacing DC-Fe_3_O_4_ with conventional surfactant sodium lauryl polyoxyethylene ether sulfate (AES) without a phenyl ring, the pink color did not disappear either, indicating that the AES-stabilized emulsion cannot be used to adsorb the RhB from the aqueous solution (Figure S7e). The results demonstrate that the π–π stacking between benzene in RhB and phenyl ring in DC-Fe_3_O_4_ also played important roles for the adsorption of RhB into the oil–water interface. Pickering emulsions have good magnetic responsiveness and high stability, so emulsion droplets adsorbed by RhB can be regenerated upon washing and used to extract RhB again. Even after three times, the extraction efficiency was still above 78%. During extraction, the efficiency of the extraction decreased owing to the adsorption of residual RhB molecules at the droplet interface, which were difficult to remove. Pickering emulsions can potentially serve as extraction systems for dye molecules (such as RhB) because of their high efficiency through simple, yet rapid magnetic separation. Furthermore, the Pickering emulsions could be reused for extraction by washing. This simple magnetic separation method broadens the application of magnetism-responsive Pickering emulsions.

## 4. Conclusions

To summarize, this work has developed a dual-responsive oil-in-water Pickering emulsion prepared using dynamic covalent Fe_3_O_4_ (DC-Fe_3_O_4_) as a stabilizer. At pH 10, the hydrophobic functionalization with benzaldehyde through dynamic imine bond (DIB) allowed DC-Fe_3_O_4_ nanoparticles to reach the oil–water interface, forming stable Pickering emulsions. By reducing the pH from 10 to 2, DBI is decomposed so that amphiphilic DC-Fe_3_O_4_ breaks into highly hydrophilic Fe_3_O_4_-NH_2_ and surface-inactive benzaldehyde, resulting in a phase separation of the Pickering emulsion. Besides, Pickering emulsion stabilized by DC-Fe_3_O_4_ nanoparticles was also magnetically responsive. The magnetism-responsive Pickering emulsion could be used for the extraction of RhB-polluted aqueous solutions with a high extraction efficiency, and Pickering emulsion droplets can be used for extraction at least three times after washing, thanks to their magnetic responsiveness and the high stability of Pickering emulsion. The feasible and unique dual-responsiveness enrich the intelligent control of Pickering emulsions’ stability and broaden the applications of Pickering emulsion in field wastewater treatments.

## Supplementary Materials

The following supporting information can be downloaded at https://www.mdpi.com/article/10.3390/nano12152587/s1, Figure S1. TEM image of DC-Fe_3_O_4_, Figure S2. Photograph of DC-Fe_3_O_4_ nanoparticles partitioning at oil-water interface. The aqueous phase was stained with RbB, Figure S3. Photographs of liquid paraffin in water Pickering emulsions stabilized by 1.0 wt% Fe_3_O_4_ (a), 1.0 wt% Fe_3_O_4_-NH_2_ (b) at pH 10, taken 30 min after preparation, Figure S4. Photograph of the liquid paraffin and water after sonication for 2 min without DC-Fe_3_O_4_ nanoparticles, Figure S5. Image of contact measurement of acidic water droplet (pH = 2) on the DC-Fe_3_O_4_ film, Figure S6. Optical micrographs of initial Pickering emulsion and after 3 cycles are shown in (a) and (b), respectively. Pickering emulsion was prepared with 1.0 wt% DC-Fe_3_O_4_ at pH 10. The liquid paraffin and water were in an equal volume ratio, Figure S7. Photographs of 4 mg/L rhodamine B solution (a), the extraction of rhodamine B solution with DC-Fe_3_O_4_ nanoparticles (b), the extraction rhodamine B solution with liquid paraffin (c), extraction of rhodamine B solution with DC-Fe_3_O_4_ stabilized oil in water Pickering emulsion (d), and extraction of rhodamine B solution with AES stabilized oil in water emulsion (e), Figure S8. Standard curve of rhodamine B, Figure S9. The change of RhB concentration with standing time after adding 1 mL Pickering emulsion into 4 mL RhB aqueous solution. The initial concentration of RhB is 4 mg/L. The Pickering emulsion was stabilized by 1 wt% DC-Fe_3_O_4_ and the volume ratio of liquid paraffin and water is 1:1.
