# Supplementary material for: pH and Magnetism Dual-Responsive Pickering Emulsion Stabilized by Dynamic Covalent Fe3O4 Nanoparticles
Source: Nanomaterials (Basel). 2022 Jul 28;12(15):2587. doi: 10.3390/nano12152587 (PMC9370824; doi:10.3390/nano12152587)
Supplement: Supplementary file 1 [file nanomaterials-12-02587-s001.zip › nanomaterials-1807153-supplementary.pdf]

pH and Magnetism Dual-Responsive Pickering Emulsion Stabilized by Dynamic Covalent Fe_3O_4 Nanoparticles

Gaihuan Ren [1,*], Zhanzhao Li [1], Dongxu Lu [2], Bo Li [1], Lulu Ren [3], Wenwen Di [3], Hongqin Yu [1], Jianxin He [1], and Dejun Sun [3]

[1] Textile and Garment Industry of Research Institute, Zhongyuan University of Technology, Zhengzhou 450007, China; 2021117608@zut.edu.cn (Z.L.); 2020110366@zut.edu.cn (B.L.); 3812@zut.edu.cn (H.Y.); 5269@zut.edu.cn (J.H.)
[2] School of Mechanical and Electrical Engineering, Zhengzhou University of Industrial Technology, Zhengzhou 450007, China; tian543669@163.com
[3] Key Laboratory of Colloid and Interface Chemistry, Ministry of Education, Shandong University, Jinan 250100, China; 201912144@mail.sdu.edu.cn (L.R.); 201832281@mail.sdu.edu.cn (W.D.); djsun@zut.edu.cn (D.S.)
* Correspondence: ghren@zut.edu.cn

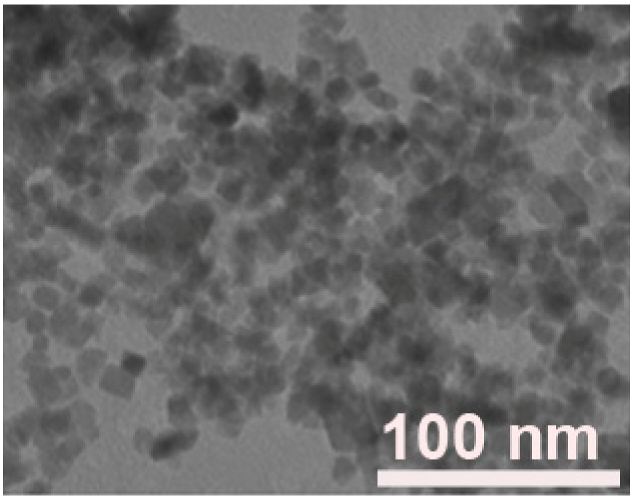

Figure S1. TEM image of DC-Fe_3O_4.

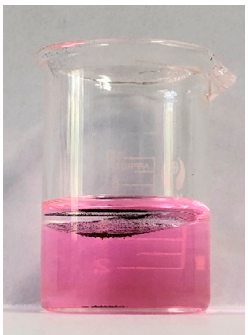

Figure S2. Photograph of DC-Fe_3O_4 nanoparticles partitioning at oil-water interface. The aqueous phase was stained with RbB.

.

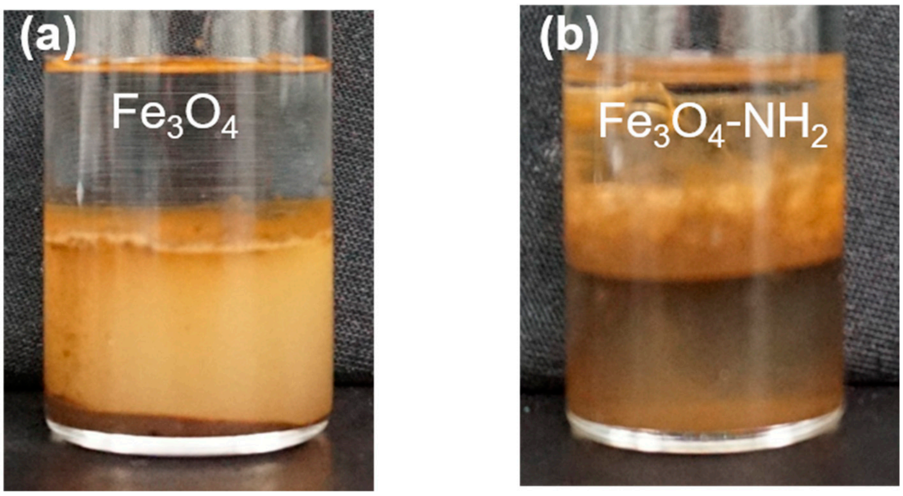

Figure S3. Photographs of liquid paraffin in water Pickering emulsions stabilized by 1.0 wt% Fe_3O_4 (a), 1.0 wt% Fe_3O_4-NH_2 (b) at pH 10, taken 30 min after preparation.

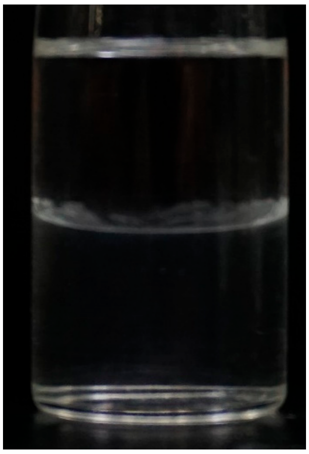

Figure S4. Photograph of the liquid paraffin and water after sonication for 2 min without DC-Fe_3O_4 nanoparticles.

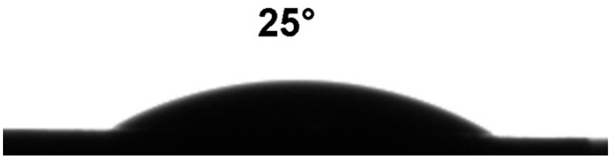

Figure S5. Image of contact measurement of acidic water droplet (pH = 2) on the DC-Fe_3O_4 film.

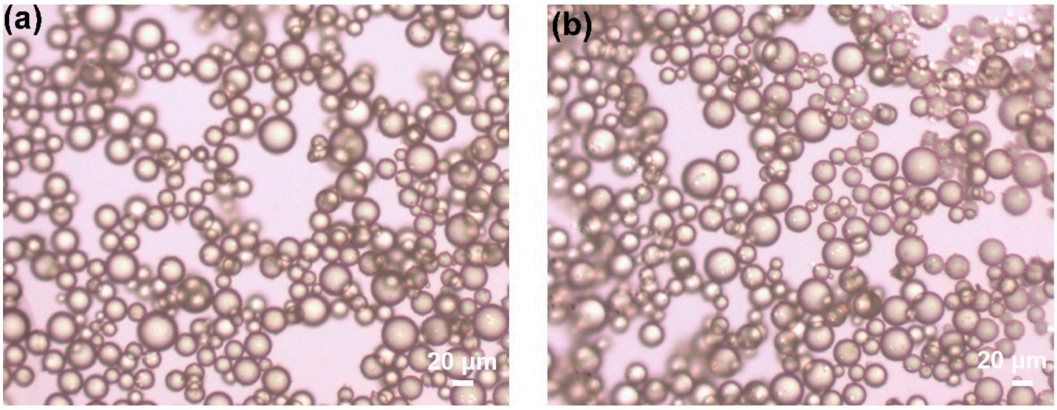

Figure S6. Optical micrographs of initial Pickering emulsion and after 3 cycles are shown in (a) and (b), respectively. Pickering emulsion was prepared with 1.0 wt% DC-Fe3O4 at pH 10. The liquid paraffin and water were in an equal volume ratio.

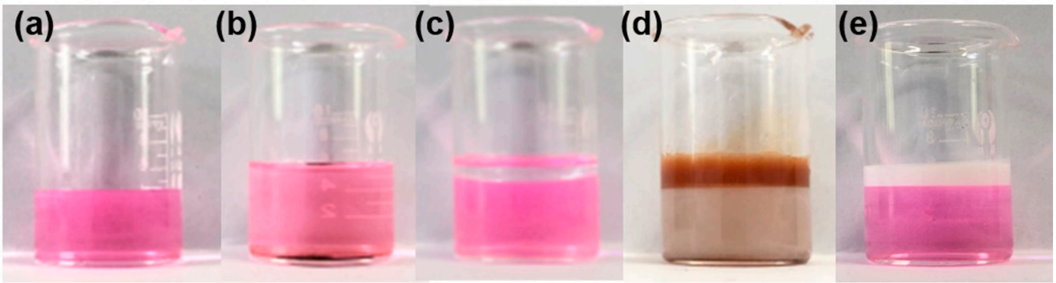

Figure S7. Photographs of 4 mg/L rhodamine B solution (a), the extraction of rhodamine B solution with DC-Fe_3O_4 nanoparticles (b), the extraction rhodamine B solution with liquid paraffin (c), extraction of rhodamine B solution with DC-Fe_3O_4 stabilized oil in water Pickering emulsion (d), and extraction of rhodamine B solution with AES stabilized oil in water emulsion (e).

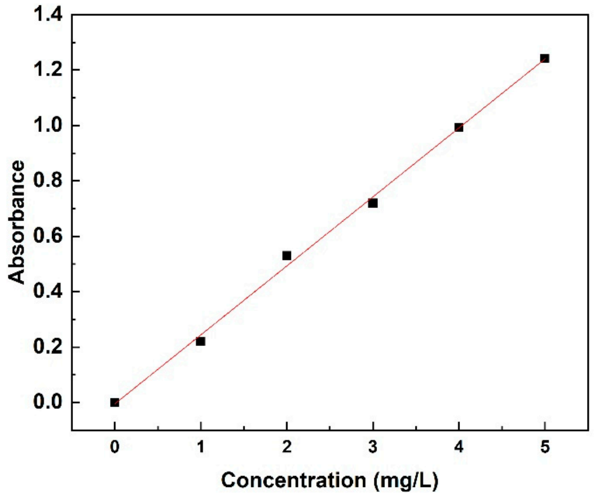

Figure S8. Standard curve of rhodamine B.

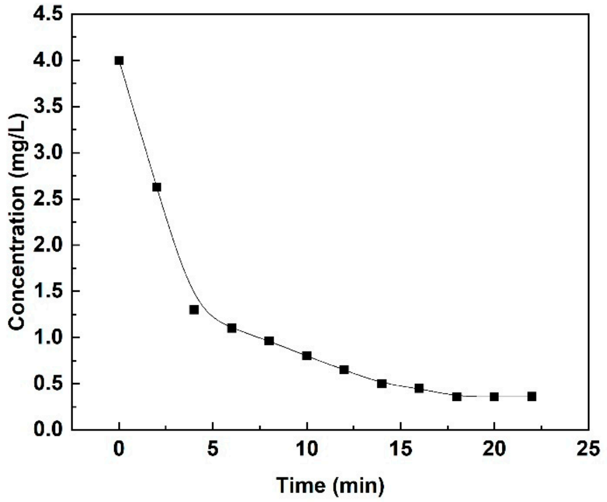

Figure S9. The change of RhB concentration with standing time after adding 1 mL Pickering emulsion into 4 mL RhB aqueous solution. The initial concentration of RhB is 4 mg/L. The Pickering emulsion was stabilized by 1 wt% DC-Fe_3O_4 and the volume ratio of liquid paraffin and water is 1:1.
